# cPLA2 activation contributes to lysosomal defects leading to impairment of autophagy after spinal cord injury

**DOI:** 10.1038/s41419-019-1764-1

**Published:** 2019-07-11

**Authors:** Yun Li, Jace W. Jones, Harry M. C. Choi, Chinmoy Sarkar, Maureen A. Kane, Eugene Y. Koh, Marta M. Lipinski, Junfang Wu

**Affiliations:** 1 Department of Anesthesiology & Center for Shock, Trauma and Anesthesiology Research (STAR), School of Medicine, University of Maryland, Baltimore, MD USA; 2 Department of Pharmaceutical Sciences, School of Pharmacy, University of Maryland, Baltimore, MD USA; 3 Department of Orthopedics, School of Medicine, University of Maryland, Baltimore, MD USA

**Keywords:** Cell death in the nervous system, Spinal cord diseases

## Abstract

The autophagy–lysosomal pathway plays an essential role in cellular homeostasis as well as a protective function against a variety of diseases including neurodegeneration. Conversely, inhibition of autophagy, for example due to lysosomal dysfunction, can lead to pathological accumulation of dysfunctional autophagosomes and consequent neuronal cell death. We previously reported that autophagy is inhibited and contributes to neuronal cell death following spinal cord injury (SCI). In this study, we examined lysosomal function and explored the mechanism of lysosomal defects following SCI. Our data demonstrated that expression levels and processing of the lysosomal enzyme cathepsin D (CTSD) are decreased by 2 h after SCI. Enzymatic activity levels of CTSD and another lysosomal enzyme, N-acetyl-alpha-glucosaminidase, are both decreased 24 h post injury, indicating general lysosomal dysfunction. Subcellular fractionation and immunohistochemistry analysis demonstrated that this dysfunction is due to lysosomal membrane permeabilization and leakage of lysosomal contents into the cytosol. To directly assess extent and mechanisms of damage to lysosomal membranes, we performed mass spectrometry-based lipidomic analysis of lysosomes purified from SCI and control spinal cord. At 2 h post injury our data demonstrated increase in several classes of lysosophospholipids, the products of phospholipases (PLAs), as well as accumulation of PLA activators, ceramides. Phospholipase cPLA2, the main PLA species expressed in the CNS, has been previously implicated in mediation of secondary injury after SCI, but the mechanisms of its involvement remain unclear. Our data demonstrate that cPLA2 is activated within 2 h after SCI preferentially in the lysosomal fraction, where it colocalizes with lysosomal-associated membrane protein 2 in neurons. Inhibition of cPLA2 in vivo decreased lysosomal damage, restored autophagy flux, and reduced neuronal cell damage. Taken together our data implicate lysosomal defects in pathophysiology of SCI and for the first time indicate that cPLA2 activation leads to lysosomal damage causing neuronal autophagosome accumulation associated with neuronal cell death.

## Introduction

During traumatic spinal cord injury (SCI) some neurons are directly mechanically damaged, but many others die as a result of injury-induced biochemical changes (secondary injury)^1–5^. Thus, blocking or attenuating secondary neuronal damage could significantly limit incapacitation consequent to injury. However, underlying mechanisms of secondary neuronal cell death after SCI remains incomplete.

Autophagy is a lysosome-dependent catabolic pathway that functions to degrade cytoplasmic proteins, protein aggregates and organelles, and is essential in maintaining cellular homeostasis and protection from neurodegeneration^6,7^. However, when lysosomal function is compromised autophagy can also contribute to cell death. Autophagic dysregulation has been implicated as one of the major causes of neuronal cell death in several neurodegenerative diseases such as Alzheimer and Parkinson disease^8–10^. Our prior studies^11^ demonstrated accumulation of autophagosomes in neuronal cell bodies after SCI, which is due to impairment of autophagy flux. This is likely caused by lysosomal dysfunction, as evidenced by lower protein levels and enzymatic activity of the lysosomal enzyme, cathepsin D (CTSD) in injured spinal cord immediately after injury^12^. SCI-mediated block of autophagy flux is associated with increased endoplasmic reticulum (ER) stress and both apoptotic and necroptotic neuronal cell death^11,12^. However, the mechanisms leading to inhibition of the autophagy–lysosomal pathway in neuronal injury after SCI remain unknown.

We showed previously^11^ that SCI also alters intracellular localization of CTSD—diffuse rather than discrete punctate, suggesting the possibility that lysosomal membrane permeabilization (LMP) allows leakage of CTSD into cytosol, resulting in decreased lysosomal activity and inhibition of autophagy flux after SCI. Thus, preservation of lysosomal membrane integrity is of utmost importance not only for maintenance of lysosomal function but also to protect cellular components from exposure to lysosomal luminal enzymes. Yet, the mechanisms by which the lysosomal lipid membrane is altered under pathological conditions remain poorly understood.

Lysosomes are surrounded by a phospholipid containing membrane, making them vulnerable to the activation of PLAs. There are three major PLAs present in the central nervous system: calcium dependent secretory phospholipase A2 (sPLA2), cytosolic phospholipase A2 (cPLA2), as well as calcium independent phospholipase A2 (iPLA2)^13,14^. Among these, cPLA2 is considered to be the most important PLA2 isoform, because it has been implicated as an effector in receptor-mediated release of arachidonic acid (AA) and exhibits strong preference for deacylation of AA over other fatty acids^15,16^. cPLA2 levels and activity are increased after SCI and contribute to neuronal cell death^17,18^. Blocking cPLA2 pharmacologically and genetically reduces tissue damage and improves motor functional recovery after SCI^18,19^. However, its mechanisms are not fully understood. It has been reported that cPLA2 may directly cause loss of membrane integrity^20^ and may participate in LMP in vitro^21–23^. As cPLA2 can damage cellular membranes, we hypothesized that it may be involved in LMP after SCI.

Because lysosomal function is necessary to support autophagy flux, the aims of this study were to: (1) examine lysosomal function and explore the mechanism of lysosomal defect following SCI; and (2) investigate if cPLA2 participates in lysosomal damage and inhibition of autophagy flux after SCI. Our results show lysosomal membrane damage in injured spinal cord that is correlated with increased activation of cPLA2. Mass spectrometry (MS)-based lipidomics demonstrates increase in several classes of lysosophospholipids, the products of PLAs, as well as accumulation of PLA activators, ceramides in lysosomes purified from SCI spinal cord. Early pharmacological inhibition of cPLA2 in vivo reduces lysosomal damage and restores autophagy flux, leading to reduced neuronal cell damage following SCI.

## Results

### Autophagy flux is inhibited after SCI at the level of lysosomes

**Fig. 1:**
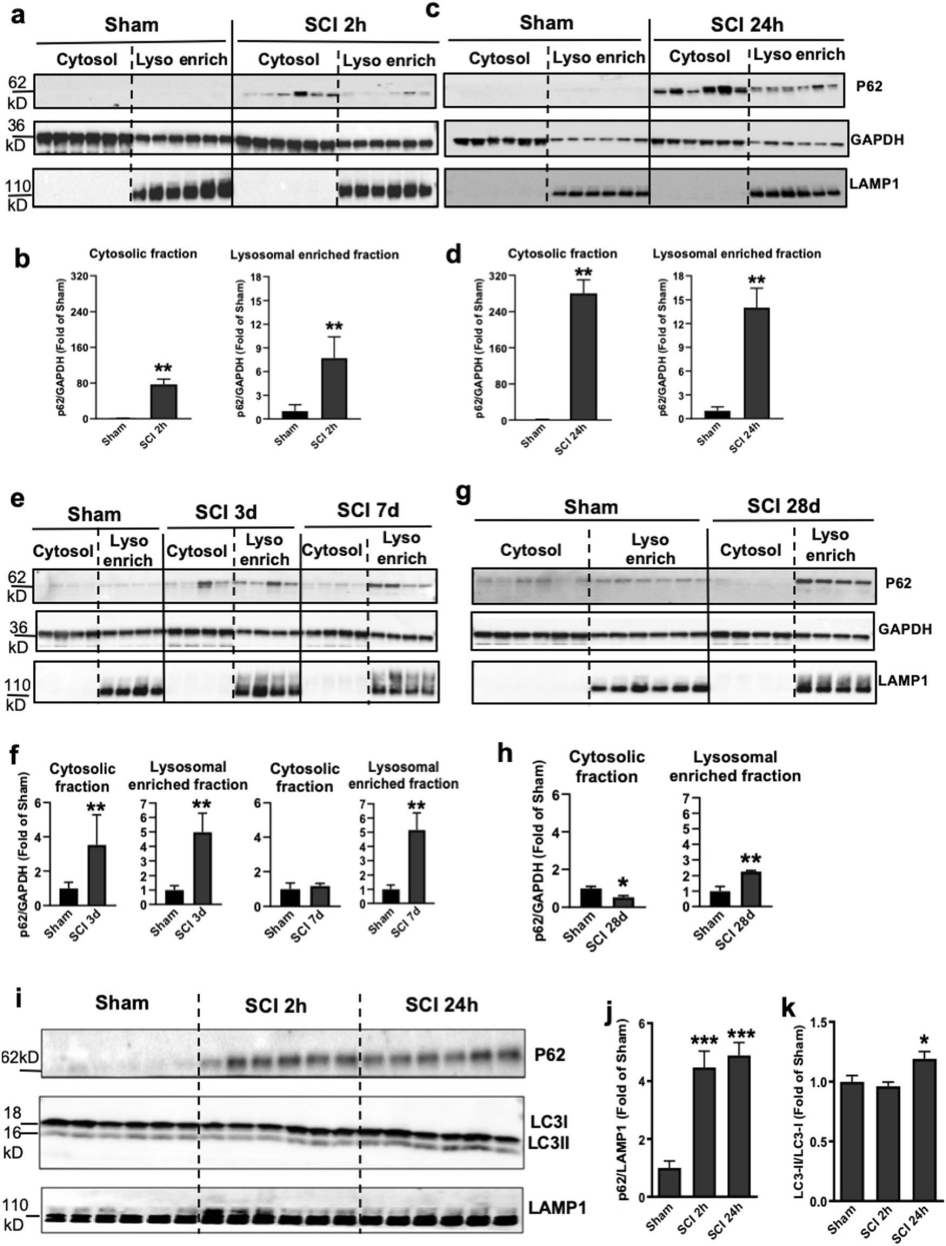
SCI causes impaired autophagy flux at the level of lysosomes. **a**–**h** Time course of the adapter protein p62 (SQSTM1) expression in the cytosol and at the lysosome-enriched fractions derived from spinal cord tissue surrounding injury site following SCI in mice. Spinal cord samples were collected at indicated time points, fractionated to isolate cytosolic and lysosome-enriched fractions, processed for western blot and blotted with indicated antibodies. Lysosomal membrane protein LAMP1 was used to identify lysosomal fraction and as a loading control. All data are presented as mean ± SEM. Mann–Whitney test (two-tailed). *n* = 6 from 12 mice/group. *p < 0.05, **p < 0.01 versus Sham. **i**–**k** Expression of p62 and the autophagosome marker LC3-II at purified lysosomes from sham control and injured spinal cords at 2 and 24 h. All data are presented as mean ± SEM. One-way ANOVA, Tukey post hoc analysis. *n* = 6 from 12 mice/group. *p < 0.05, ***p < 0.001 versus Sham

We previously demonstrated that SCI leads to inhibition of autophagy in contusion SCI^11^. To identify the stage of the autophagy pathway disrupted after SCI, we isolated lysosome/autolysosome-enriched fractions^24^ from sham control and injured spinal cords at 2 h and 1, 3, 7, 28 days post injury. Quantitative analysis of Western blot showed that the adapter protein p62 (SQSTM1), which mediates delivery of ubiquinated cargo to autophagosomes, rapidly increases in both the cytosol and lysosome-enriched fractions at 2 h after injury compared with sham tissue, remaining elevated in lysosomal fraction up to 28 days after SCI (Fig. 1a–h). To confirm the association of autophagosome markers with the lysosomes after SCI, we used a more stringent isolation method to obtain purified lysosomes^24^ from sham control and injured spinal cords at 2 and 24 h. Western blot data showed rapid accumulation of p62/SQSTM1 protein starting at 2 h after injury and continuing through 24 h post injury (Fig. 1i–k). This was accompanied by an accumulation of the autophagosome marker LC3-II at 24 h post injury, indicating inhibition of autophagy flux at the level of the lysosomes.

### SCI leads to lysosomal membrane permeabilization (LMP)

**Fig. 2:**
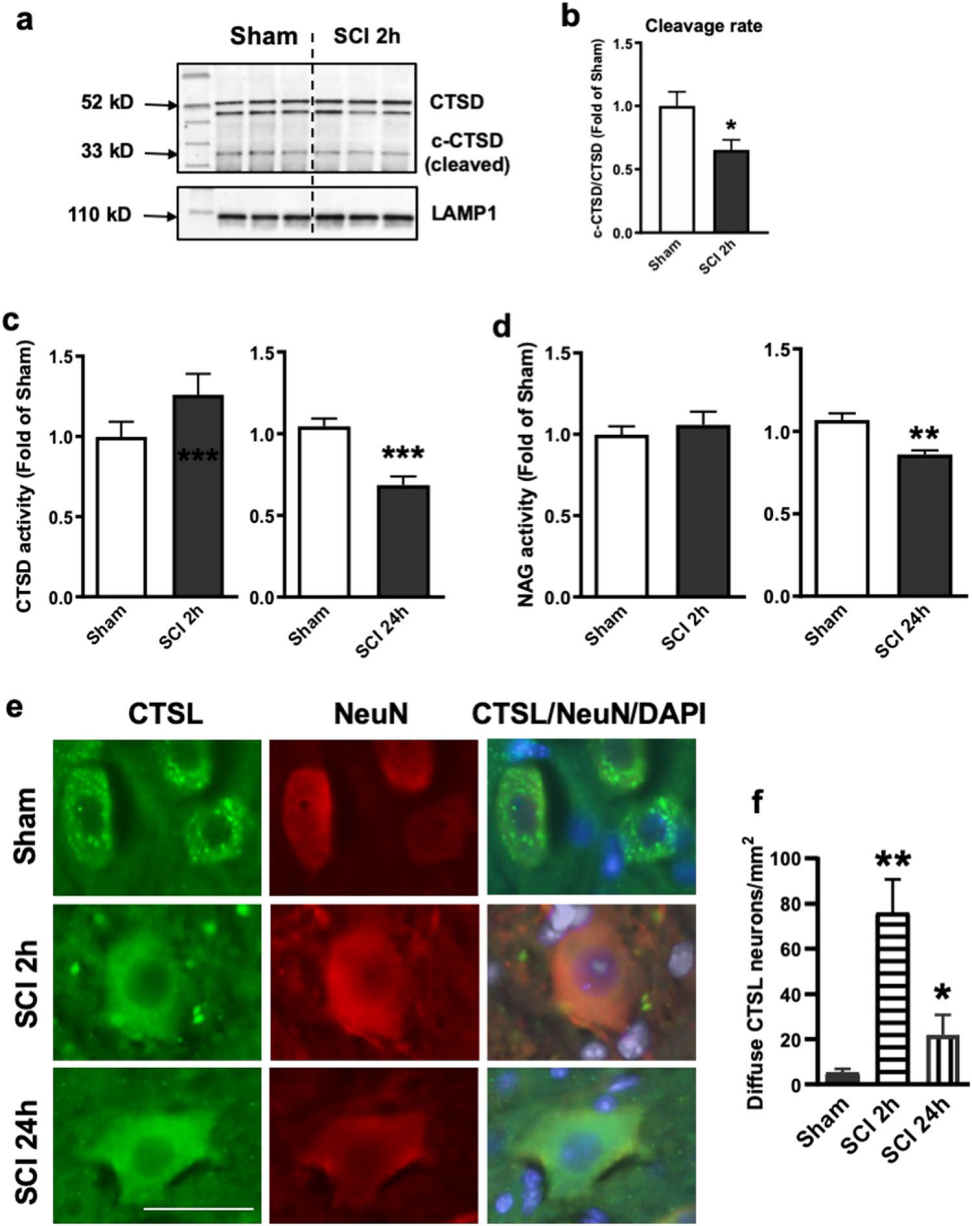
SCI causes an increase in lysosomal membrane permeability. **a**, **b** Expression of cathepsin D (CTSD) in the purified lysosomes from sham control and injured spinal cords at 2 h. Dissected spinal cord tissue was purified for lysosomes, then processed for western blot. Both full length precursor and cleaved active CTSD are indicated in **a**. Lysosomal membrane protein LAMP1 was used to identify lysosomal fraction and as a loading control. Quantification of cleavage rate (cleaved/precursor CTSD) is indicated in **b**. All data are presented as mean ± SEM. Mann–Whitney test (two-tailed). *n* = 6 mice/group. *p < 0.05 versus Sham. **c**, **d** Activity of lysosomal enzymes **c** CTSD and **d** N-acetyl-glucosaminidase (NAG) is decreased in purified lysosomes from sham and SCI mouse spinal cord at 24 h. Data are mean ± SEM, Mann–Whitney test (two-tailed), *n* = 6 mice/group; **p < 0.01, ***p < 0.001 versus Sham. **e**, **f** Images (20×) demonstrating diffused soluble lysosomal enzyme cathepsin L (CTSL, green) in spinal cord ventral horn neurons (NeuN, red) at 2 and 24 h after SCI. CTSL staining appeared punctate in sham mouse spinal cord ventral horn neurons. Quantification of neurons with diffused CTSL staining is indicated in **f**. Data are mean ± SEM, Unpaired *t-*test between two groups, *n* = 5 mice (sham, SCI 2 h) and 3 mice (SCI 24 h). *p < 0.05, **p < 0.01 versus Sham. Scale bar = 50 μm

Next, we purified lysosomes from sham control and injured spinal cords to examine the effects that SCI might have on lysosome function by assessing the protein expression level of CTSD and the activity of several known lysosome enzymes. We observed decrease in both precursor and cleaved CTSD protein levels at the lysosomes at 2 h after SCI (Fig. 2a, b), which reflects a decrease in the amount of active CTSD that exists within the lysosome, suggesting lysosome dysfunction. Unlike CTSD, levels of lysosomal membrane proteins, lysosomal-associated membrane protein 1 (LAMP1), did not change at 2 h after SCI as compared with sham, suggesting that the size of the lysosomal compartment was not altered at that time. Furthermore, the enzymatic activity assay at purified lysosomes confirmed decreased CTSD activity at 24 h after SCI (Fig. 2c). Similar decline was observed for another lysosomal enzyme N-acetyl-alpha-glucosaminidase (NAG) (Fig. 2d), consistent with our hypothesis that SCI leads to rapid decrease in lysosomal function, thus causing inhibition of autophagy flux. We did not observe significant difference of CTSD and NAG activity at 2 h post injury (Fig. 2c, d).

Presence of lysosomal enzyme in the cytosol was confirmed by immunohistochemistry (IHC) staining spinal cord sections from sham and 2 and 24 h injured mice with antibody against soluble lysosomal enzyme cathepsin L (CTSL). We observed punctate intracellular localization of CTSL in both sham and SCI neurons, confirming that lysosomal inhibition is not due to complete destruction of neuronal lysosomes. At 2 and 24 h after injury, quantification of IHC showed increased diffuse CTSL cells in ventral horn neurons (Fig. 2e, f), consistent with our hypothesis that leakage of soluble lysosomal contents leads to loss of lysosome function in neurons after SCI.

### SCI disrupts lipid compositions of lysosomal membranes

**Fig. 3:**
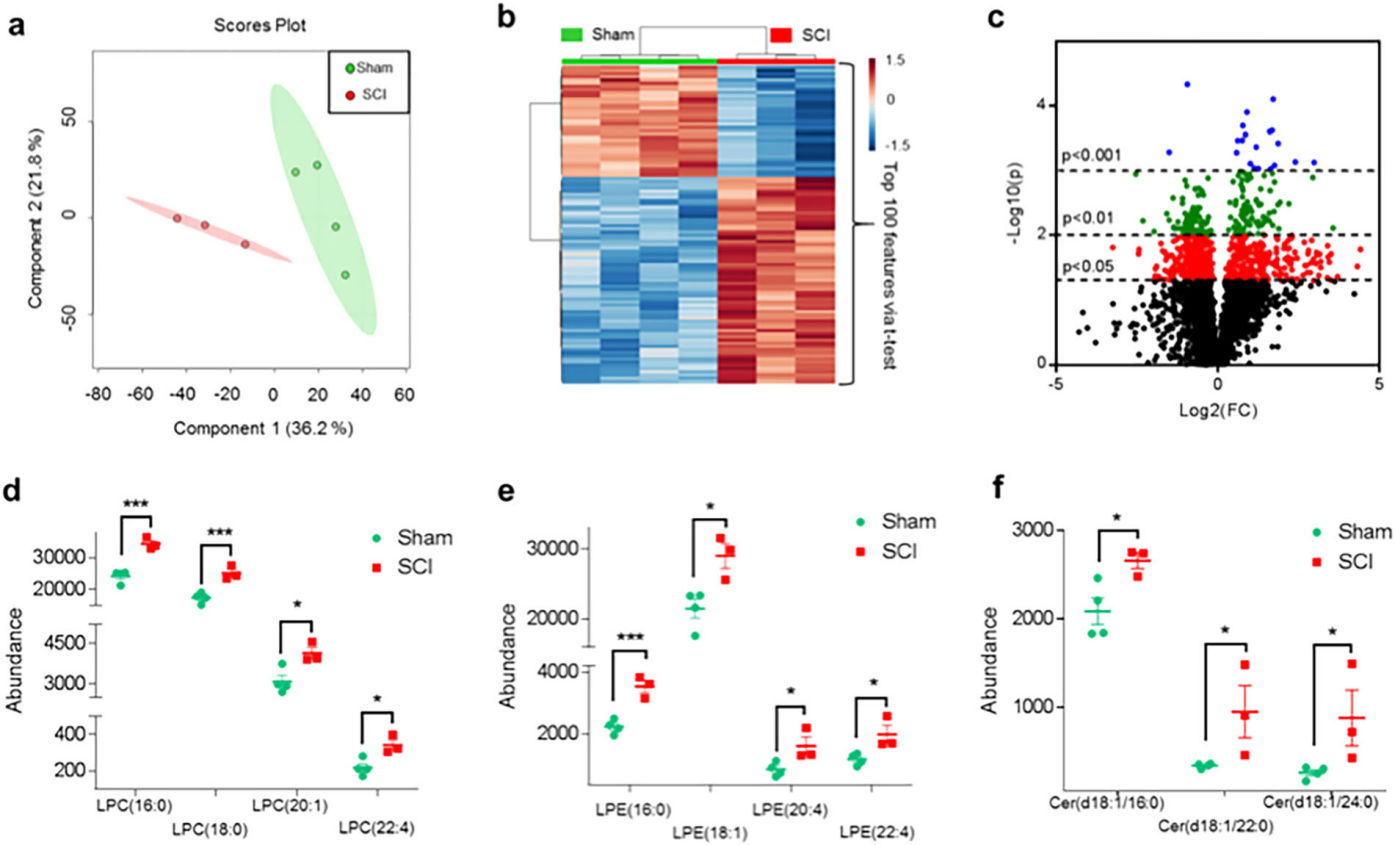
SCI alters lysosomal membrane lipid composition. Results of LC-MS/MS lipid analysis of purified lysosomes from SCI and sham spinal cord membranes at 2 h after injury. **a** Partial Least Squares-Discriminate Analysis (PLS-DA) plot comparing sham (green) and SCI (red) in positive ion mode UPLC-HDMS^E^ demonstrating separation of sham and SCI data; *R*^2^ = 0.88, *Q*^2^ = 0.51. Each point represents a data set from an individual animal. The 95% confidence intervals are indicated by elliptical patterns per group. Data were sum normalized, log transformed, and mean centered. **b** Heatmap displaying the top 100 differential abundance features based on *t*-test/ANOVA, Euclidean distancing and Ward clustering in positive ion mode UPLC-HDMS^E^. **c** Volcano plot highlighting features that had a *p < 0.05 (red), **p < 0.01 (green), and ***p < 0.001 (blue) when comparing Sham to SCI. The *x*-axis is log2(FC) (FC = fold change) and the *y*-axis is –log10(*p*) (*p* = *p*-value based on *t*-test). Plots in **a**–**c** generated using MetaboAnalyst; *n* = 4 for sham and *n* = 3 for SCI. **d**–**f** Altered abundance of specific lipid classes in lysosomal membranes from sham (green) and SCI (red) mice. Statistical significance was determined using *t*-test. **d** LPC (lysophosphatidylcholine) abundance. Calculated *p*-values were 0.0015 (LPC(16:0)), 0.0029 (LPC(18:0)), 0.0246 (LPC(20:1)), and 0.0207 (LPC(22:4)). **e** LPE (lysophosphatidylethanolamine) abundance. Calculated *p*-values were 0.0019 (LPE(16:0)), 0.0239 (LPE(18:1)), 0.0301 (LPE(20:4)), and 0.0196 (LPE(22:4)). **f** Cer (ceramide) abundance. Calculated *p*-values were 0.0395 (Cer(d18:/16:0)), 0.0240 (Cer(d18:/22:0)), and 0.0247 (Cer(d18:/24:0)). Individual data points and mean ± SEM are indicated. *n* = 4 for sham and *n* = 3 for SCI

To directly assess the extent of changes and elucidate the mechanisms of damage to lysosomal membranes, we analyzed the lipid composition of lysosomal membranes purified from sham and injured spinal cord at 2 and 24 h post injury using liquid chromatography-tandem mass spectrometry (LC-MS/MS). The total lipid extract of the lysosomal preparation was subjected to LC-MS/MS analysis (Schematically depicted in Fig. S1a). Our preparation was highly enriched in lysosomes/lysosomal content with almost undetectable levels of endoplasmic reticulum or mitochondrial proteins^25^ (Fig. S1b). Our data demonstrate significant changes in the overall lipid composition of the lysosomal preparations from SCI versus sham spinal cord, as visualized by multivariate and univariate analyses (Fig. 3a–c). Interestingly, we observed the most pronounced differences in lysosomal lipid composition between sham versus the 2-h SCI samples (Partial Least Squares-Discriminant Analysis (PLS-DA), *Q*^2^ = 0.51, Fig. 3a), rather than between sham versus 24-h SCI (PLS-DA, *Q*^2^ = 0.28) (data not shown). Since our data indicate that defects in autophagy and lysosomal function also occur very early after SCI, we focused our analysis on the 2-h time point. In total we were able to identify 87 specific lipids that differed in abundance between lysosomes from sham versus 2-h SCI spinal cord (Table S1). Strikingly, this included a significant increase in several classes of lysophospholipids (lysophosphatidylcholine (LPC) and lysophosphatidylethanolamine (LPE)) (Fig. 3d, e), the products of PLAs, as well as accumulation of PLA activators, ceramides (Fig. 3f). Therefore, the increased abundance of lysophospholipids and ceramides in the lysosomal preparations from injured spinal cord strongly suggested that lysosomal membrane damage after SCI may be mediated by the activation of PLAs.

### cPLA2 is activated and present at lysosomes after SCI

**Fig. 4:**
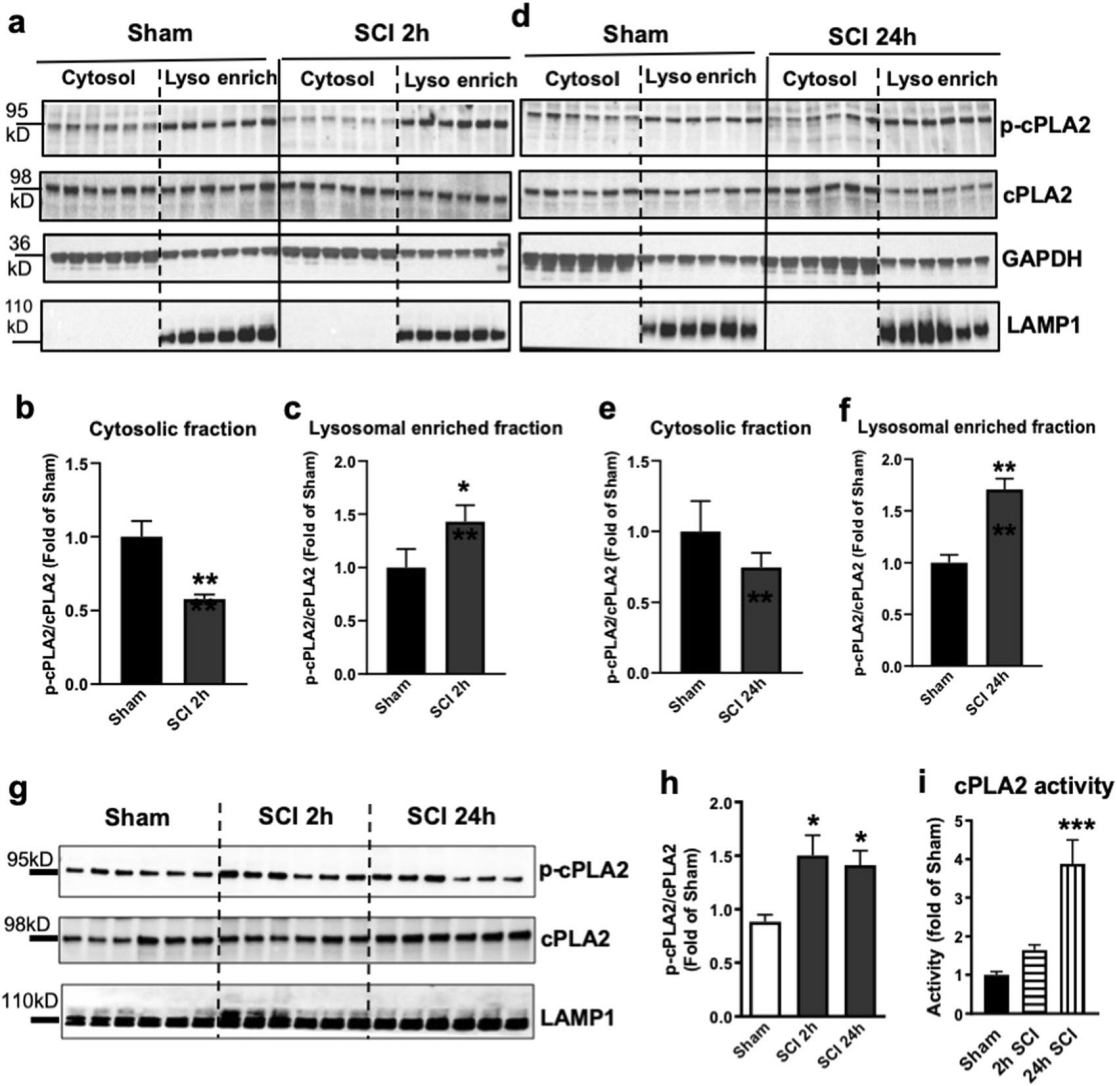
cPLA2 is activated and present at lysosomal membranes after SCI. **a**–**f** The protein expression of activated (phosphorylated, p-cPLA2) and total cPLA2 in the cytosol and the lysosome-enriched fractions isolated from sham control and injured spinal cords at 2 and 24 h post injury. Each lane represents an individual animal. Quantitative analysis of Western blot for the phosphorylation rate of cPLA2 (i.e., ratio of p-cPLA2/cPLA2) are indicated in **b**, **c**, **e**, and **f**. Data are mean ± SEM, Mann–Whitney test (two-tailed), *n* = 6 from 12 mice/group. *p < 0.05, **p < 0.01 versus Sham. **g**, **h** Expression of p-cPLA2 and cPLA2 at purified lysosomes from sham control and injured spinal cords at 2 and 24 h. Data are mean ± SEM, One-way ANOVA, Tukey post hoc analysis. *n* = 6 from 12 mice/group. *p < 0.05 versus Sham. **i** cPLA2 enzymatic activity assay was performed in the purified lysosomes at 2 and 24 h after SCI. Data are mean ± SEM, One-way ANOVA with Tukey post hoc analysis, *n* = 6 mice/group; **p < 0.001

The calcium-dependent phospholipase cPLA2 is upregulated and activated following SCI in mouse and rat models and implicated as a mediator of secondary injury^17–20^. However, the mechanisms how cPLA2 activation contributes to neuronal cell death remain unclear. To determine whether activation of cPLA2 may contribute to lysosome damage after SCI, we examined the protein expression of activated (phosphorylated, p-cPLA2) and total cPLA2 in the lysosome-enriched fractions isolated from sham control and injured spinal cords at 2 and 24 h post injury. We observed that total cPLA2 activation levels increased in the cytosolic fractions at 24 h after SC. While phosphorylated cPLA2 form also increased in lysosome-enriched fractions at both 2 and 24 h after SCI, the differences did not reach statistical significance (Fig. 4a, d). However, quantitative analysis of Western blot demonstrated that the phosphorylation rate of cPLA2 (i.e., ratio of p-cPLA2/cPLA2) was significantly increased specifically in the lysosomal fraction at 2 h after injury and persisted through 24 h after SCI (Fig. 4a–f). To confirm increased phosphorylation of cPLA2 at the lysosomes after SCI, we prepared purified lysosomes from sham control and injured spinal cords at 2 and 24 h. Western blot data showed rapid accumulation of p-cPLA2 starting at 2 h after injury, remained elevated through 24 h post injury (Fig. 4g, h). Furthermore, the enzymatic activity assay at purified lysosomes confirmed increased cPLA2 activity in the lysosomal fractions at 2 and 24 h after SCI (Fig. 4i). These data suggest that cPLA2 specifically localizes and is activated at the lysosomes after SCI, where it may directly mediate lysosomal membrane injury.

**Fig. 5:**
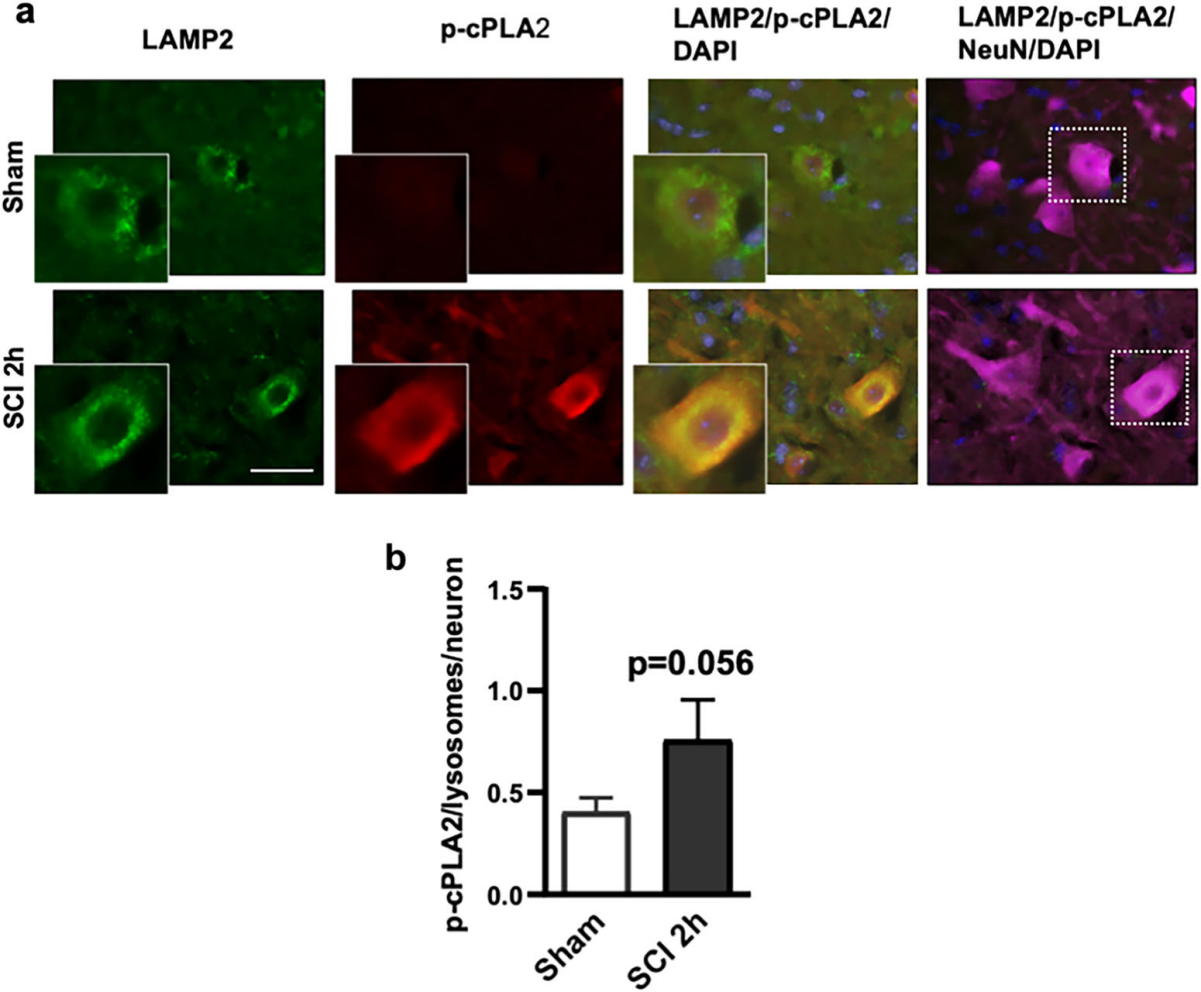
Activated cPLA2 is present at lysosomal membranes after SCI. **a** Images (20×) of neurons (NeuN, pink) in the spinal cord of sham and SCI (2 h) mice stained with antibodies against lysosomal membrane protein Lamp2 (green) and phospho-cPLA2 (red). Scale bar = 50 μm. **b** Quantification of data from **a** demonstrating increased levels of phospho-cPLA2 at lysosomes in the injured spinal cord ventral horn neurons as compared with sham. Data are presented as mean ± SEM, *n* = 8 sham and 3 SCI mice; *p* = 0.056 (Students’ *t*-test)

Using immunofluorescence labeling, we were able to observe increased p-cPLA2 expression in the ventral horn at 2 h after SCI (Fig. 5a, b). Elevated level of p-cPLA2 was observed within neurons. Moreover, this was supported by IHC analysis where we observed higher level of colocalization of phospho-cPLA2 with lysosomal marker LAMP2 in the injured spinal cord. Thus, cPLA2 is activated in mouse spinal cord ventral horn neurons after injury and is present at the lysosomes, suggesting potential direct involvement in cleavage of lysosomal membrane phospholipids to generate lysophospholipids, leading to lysosomal membrane damage and LMP after SCI.

### Inhibition of cPLA2 decreases lysosomal damage, restores autophagic flux, and is associated with reduced neuronal cell damage

**Fig. 6:**
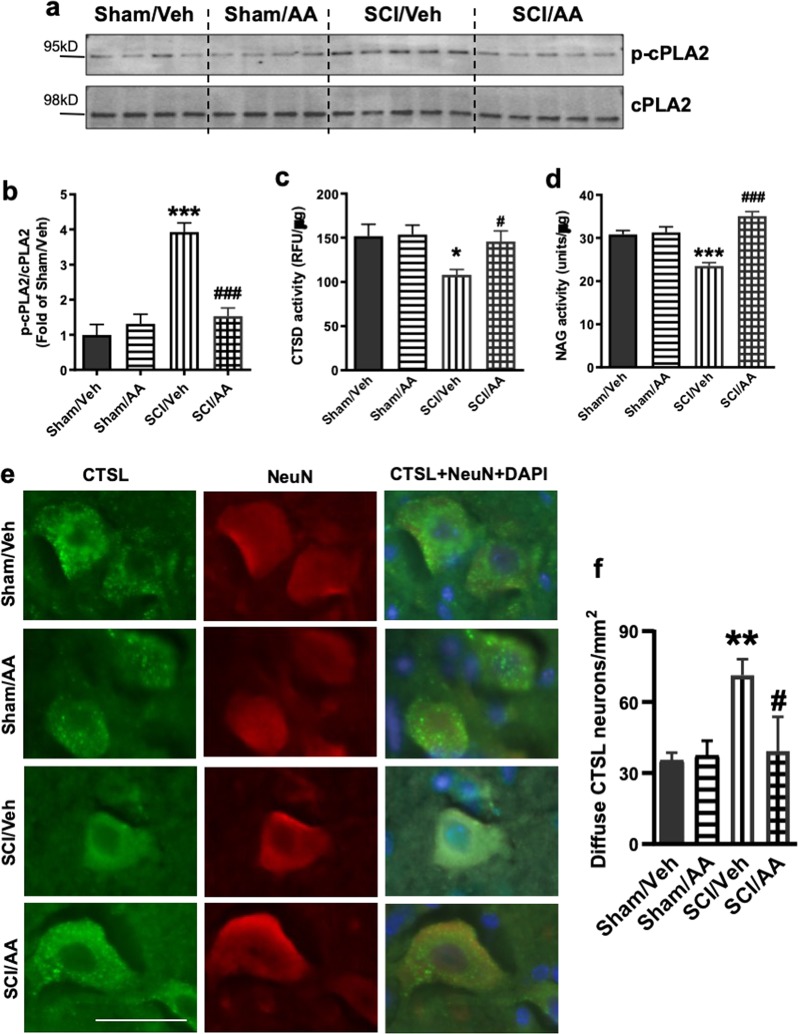
Inhibition of cPLA2 attenuates SCI-induced lysosomal membrane damage. **a**, **b** Expression of p-cPLA2 and cPLA2 at purified lysosomes from sham control and injured spinal cords at 2 and 24 h. Quantitative analysis of Western blot for the phosphorylation rate of cPLA2 (ratio of p-cPLA2/cPLA2) is indicated in **b**. *N* = 4 mice (Sham groups) and 5 mice (SCI groups). **c**, **d** Activity of lysosomal enzymes **c** CTSD and **d** N-acetyl-glucosaminidase (NAG) is increased in purified lysosomes from the injured spinal cord of mice treated with AACOCF_3_ (AA). *N* = 6 mice/group; **e**, **f** IHC analysis demonstrating decreased diffused soluble lysosomal enzyme cathepsin L (CTSL, green) in spinal cord ventral horn neurons (NeuN, red) in SCI/AACOCF3 as compared with SCI/vehicle. **e** Images (20×) of spinal cord sections from Sham/Veh, Sham/AA, SCI/Veh, and SCI/AA mice stained with antibodies against neuronal marker NeuN (red) and CTSL (green). Scale bar = 50 μm. **f** Corresponding quantification of cells with diffused (cytosolic) CTSL staining. *N* = 4 (Sham/Veh), 5 (Sham/AA), 7(SCI/Veh), and 4 (SCI/AA). Data are mean ± SEM, Two-way ANOVA with Bonferroni posttests, *p < 0.05, **p < 0.01, ***p < 0.001 versus Sham/Veh, ^#^*p* < 0.05, ^###^*p* < 0.001 versus SCI/Veh

**Fig. 7:**
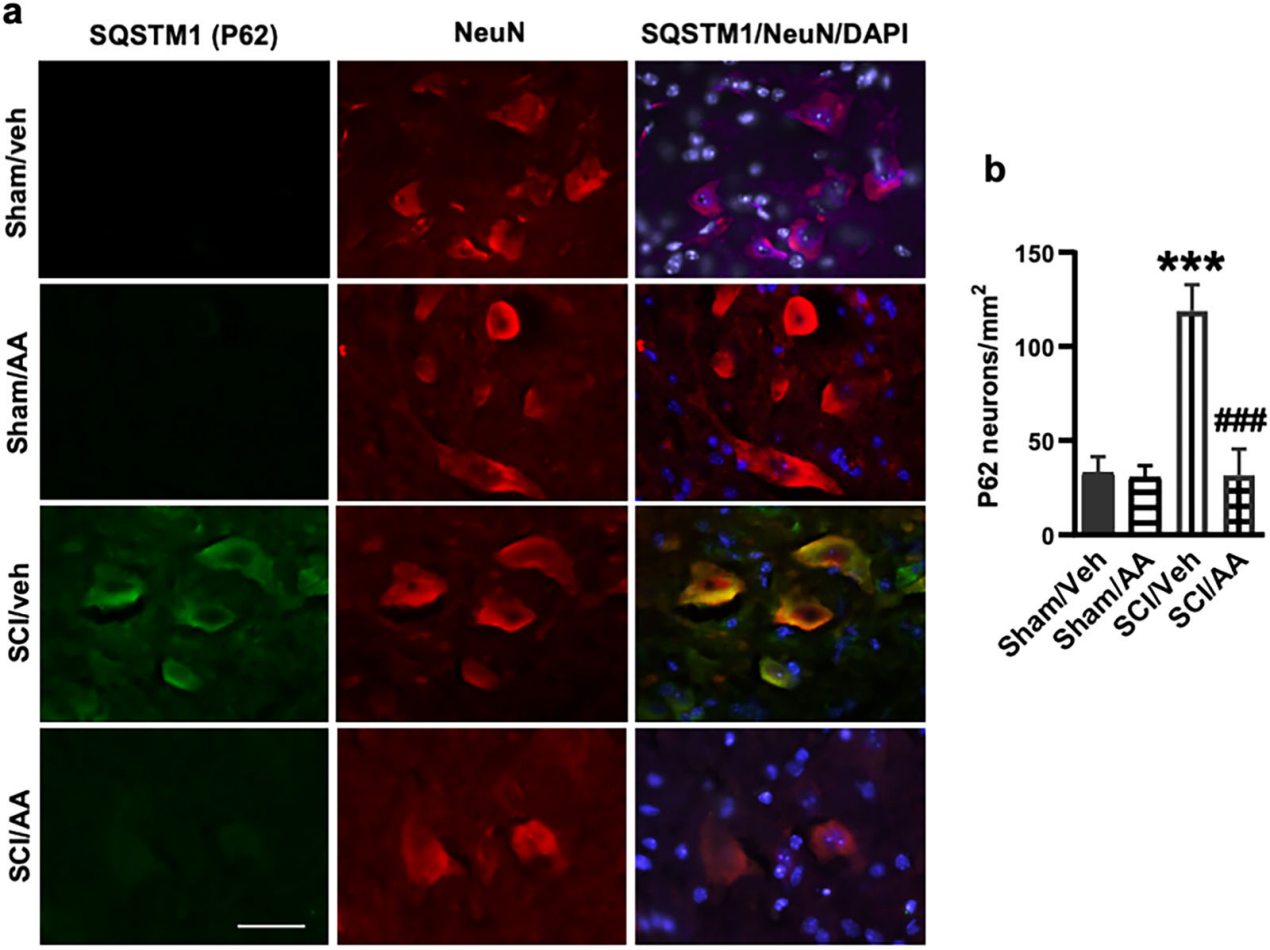
cPLA2 inhibition restores autophagy flux impaired by SCI. IHC analysis demonstrates decreased SQSTM1 (p62) accumulation in AACOCF_3_ (AA) treated SCI ventral horn neurons as compared with SCI/Vehicle group. **a** Images (20×) of spinal cord sections of Sham/Veh, Sham/AA, SCI/Veh, and SCI/AA mice stained with antibodies against neuronal marker NeuN (red) and SQSTM1 (green). Scale bar = 50 μm. **b** Quantification of SQSTM1 positive cells. Data are mean ± SEM, two-way ANOVA with Bonferroni posttests, *n* = 4 (Sham/Veh), 5 (Sham/AA), 7(SCI/Veh), and 4 (SCI/AA). ***p < 0.001 versus Sham/Veh, ^###^*p* < 0.001 versus SCI/Veh

To determine whether lysosome damage and subsequent inhibition of autophagy flux after SCI is dependent on cPLA2 activity, we treated sham and SCI mice with cPLA2 inhibitor arachidonyl trifluoromethyl ketone (AACOCF_3_). At 24 h after SCI, we detected significant reduction in cPLA2 phosphorylation in the lysosomal fractions from the AACOCF_3_-treated SCI group as compared with vehicle treated SCI controls (Fig. 6a, b), indicating inhibition of lysosomal cPLA2 activity. This was associated with increased CTSD and NAG lysosomal activity in the injured spinal cord of mice treated with AACOCF_3_ in drug-treated animals (Fig. 6c, d), suggesting restoration of lysosomal function. IHC analysis showed significantly less diffuse CTSL staining in the neuronal cytoplasm in SCI/AACOCF_3_ mice as compared with SCI/vehicle controls (Fig. 6e, f). Therefore, cPLA2 inhibition by AACOCF_3_ treatment can significantly attenuate lysosomal membrane damage and LMP after SCI. IHC analysis also showed that the treatment significantly reduced accumulation of p62 + autophagosomes in neurons at 24 h post injury (Fig. 7a, b). Together, these data suggest that cPLA2-mediated lysosomal damage was responsible for inhibition of autophagy after SCI.

**Fig. 8:**
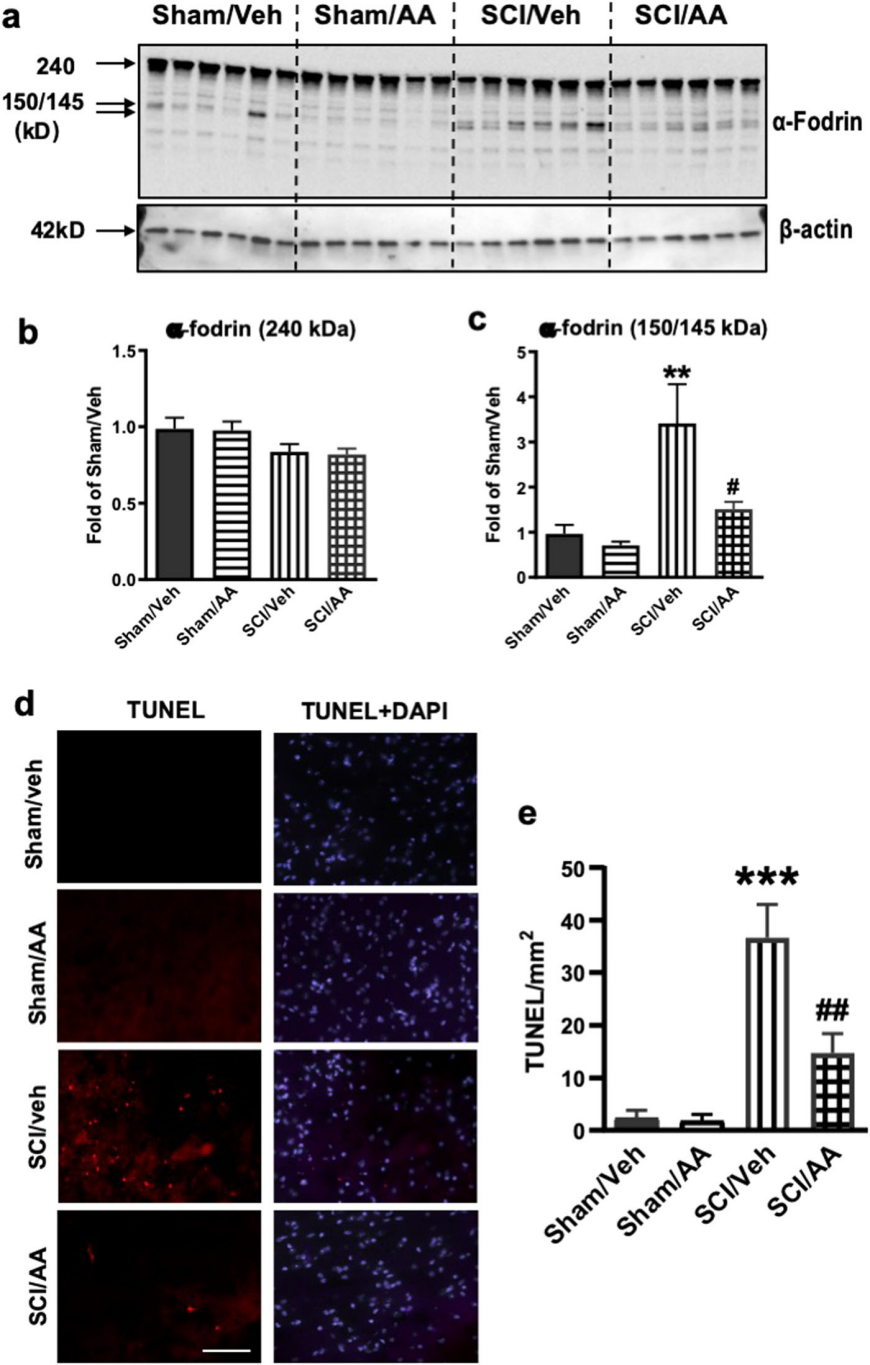
cPLA2 inhibition attenuates spinal cord ventral horn neuronal cell death in mice after SCI. **a**–**c** Expression of cleavage (145–150 kDa) and full length (240 kDa) of α-fodrin in sham and injured spinal cords at 24 h after SCI. Western blot data demonstrating decreased cleavage of α-fodrin in AACOCF3 (AA)-treated as compared with vehicle treated SCI mice. *N* = 6 mice/group. **d** Images (20×) demonstrating decreased cell death (TUNEL) in spinal cord sections from SCI/AACOCF3 as compared with SCI/Veh. Scale bar = 100 μm. **e** Quantification of TUNEL positive cells. *N* = 6 (Sham/Veh), 6 (Sham/AA), 5(SCI/Veh), and 6 (SCI/AA). Data are mean ± SEM, Two-way ANOVA with Bonferroni posttests, **p < 0.01, ***p < 0.001 versus Sham/Veh, ^#^*p* < 0.05, ^##^*p* < 0.01 versus SCI/Veh

To further investigate the neuroprotective role of cPLA2 inhibition, we examined whether decreasing cPLA2 activity by administration of AACOCF_3_ could attenuate neuronal cell damage and death after SCI. Cleavage of the marker of cellular damage, α-fodrin, was assessed in the injured spinal cord at 24 h post injury by Western blotting analysis. A total of 145/150 kDa cleavage fragments of α-fodrin were increased 3-fold, whereas lull-length protein was slightly decreased in SCI versus sham spinal cord. These changes were significantly attenuated in SCI mice treated with AACOCF_3_ (Fig. 8a–c), confirming decreased cell injury upon cPLA2 inhibition.

We previously demonstrated that disruption of autophagy is associated with neuronal cell death in the spinal cord ventral horns after SCI^11^. To determine if cPLA2 inhibition can prevent spinal cord neuronal cell death at 1 day after injury, we performed the TUNEL assay. As expected, markedly higher numbers of TUNEL positive cells were detected in all SCI tissues as compared with sham controls (Fig. 8d, e). However, the AACOCF_3_ treated SCI tissues had significantly fewer TUNEL positive cells than SCI/vehicle controls, confirming attenuation of cell death. Thus, cPLA2 inhibition by AACOCF_3_ treatment significantly restricted cell loss after SCI.

## Discussion

In this study, we demonstrate that inhibition of autophagic degradation after SCI is due to lysosomal membrane damage leading to LMP and profound lysosomal dysfunction occurring within 2 h after injury. The results of our MS-based lysosomal lipidomic analysis, demonstrating increase in several classes of lysosophospholipids, the products of PLAs, as well as accumulation of PLA activators, ceramides, strongly implicate involvement of PLAs in mediation of the lysosomal membrane damage after SCI. Furthermore, our data demonstrating accumulation and activation of cPLA2 specifically at the lysosomes, single out this PLA as the mediator of SCI-induced lysosomal membrane damage. This is further supported by the fact that inhibition of cPLA2 decreased lysosomal damage, restored autophagy flux, and reduced neuronal cell damage after SCI. Together, these data indicate that cPLA2 activation leads to lysosomal membrane damage causing autophagosome accumulation in the spinal cord and contributing to neuronal cell death after SCI.

We reported recently^11,12^ that autophagy is impaired after contusion SCI in both rat and mouse and associated with neuronal apoptosis and necroptosis. This is at least in part due to lysosomal impairment observed after SCI. Our current data further show increase of autophagy marker LC3-II and its substrate p62 in the lysosomal-enriched fractions as well as purified lysosomes, confirming that impairment of autophagy flux occurs at the level of the lysosomes. We have previously shown^11,12^ that while impairment of autophagy flux persists over time, it affects different cell types at different times. In this study, we are focusing on autophagy in neurons, which is an early cell type affected. Moreover, autophagy impairment in neurons proceeds and is correlated with the peak of neuronal cell death that is 24 h post injury^11,12^. This is also the time point at which we evaluated effects of cPLA2 on cell death in our current paper. We do not observe inhibition of autophagy in neurons at later time points. Similarly, number of TUNEL+ cells declines after the day 1 peak in the spinal cord after SCI^26–28^.

Our previous studies^11,12,24^ demonstrated that lysosomal function may be impaired at the early time points after traumatic brain and SCI, contributing to inhibition of autophagy flux. Our current data confirm that SCI causes lysosomal dysfunction, as evidenced by reduced protein levels and enzymatic activity of the lysosomal enzymes in injured spinal cord tissue as early as 2 h post injury. This suggests that lysosomal dysfunction may be one of the apical cellular events contributing to initiation, early propagation and potentiation of the secondary injury cascades. Thus, our data implicate lysosomal defects in pathophysiology of SCI. As lysosomal defects can lead to cytotoxicity in both autophagy dependent and independent manner^29^, this is likely to open up additional avenues of investigation into the importance of lysosomal damage-dependent cell death pathways in SCI. Indeed, our prior reports^11,12^ point to the crucial role of lysosomal dysfunction as an early event contributing to neuronal secondary injury through induction of apoptosis and necroptosis.

In this study, we demonstrate that contusion SCI in mice leads to neuronal LMP and use in vivo lysosomal lipidomics to suggest that this effect is mediated through the activation of cPLA2. Our MS-based lipidomics demonstrate that SCI causes alteration in lysosomal membrane lipid composition including increase in several classes of lysosophospholipids, the products of PLAs, as well as accumulation of PLA activators, ceramides. cPLA2 activation is increased in the injured spinal cord^18,19^ and has been shown to participate in induction of neuronal cell death after SCI^17,20^. Although blocking cPLA2 pharmacologically and genetically reduces neuronal cell death^18,19^, its mechanisms are not fully understood. cPLA2 is known to contribute to SCI damage via delayed mechanisms such as proinflammatory functions of some of its metabolites^20,30^. Our data indicate that the initial neuron-specific activation of cPLA2 is involved in the lysosomal membrane damage, inhibition of autophagy, and subsequent neuronal cell death. SCI-triggered early activation of cPLA2 is likely mediated by a rapid increase in intracellular calcium as well as activated upstream kinases like ERK1/2 and p38 MAPK^20,31^. LMP is implicated in many neurodegenerative conditions^32–38^, but its mechanisms are only now being elucidated. cPLA2 has been implicated as a potential LMP mediator in vitro^21–23^, but mechanisms and relevance of its involvement are not known. Our data implicate cPLA2 as a novel LMP mediator in vivo, in a disease-relevant model. cPLA2-mediated hydrolysis of membrane phospholipids can alter membrane lipid composition, straining the membrane structural integrity and leading to LMP. Lysophospholipids generated following cPLA2-mediated hydrolysis of membrane phospholipids have detergent-like properties and can directly lead to pore formation^39^.

Systemic administration of cPLA2 inhibitor AACOCF_3_ prevents lysosomal damage, attenuates inhibition of autophagy, and leads to reduced neuronal cell death after SCI. Thus, improved motor functional recovery previously observed in cPLA2 inhibitor-treated animals after SCI^18^ may be resulted, at least in part, from restored lysosomal function. It has been previously^18,19^ reported that inhibition or ablation of cPLA2 reduces neuronal loss at 24 h post injury, which is associated with improved functional recovery after SCI. However, cPLA2 is also expressed by other cell types in the spinal cord^18–20^ and effects of its inhibition on functional recovery likely reflect broader effects than just improved neuronal survival. In this study, we specifically focus on function of cPLA2 in lysosomal damage and inhibition of autophagy and their effects on neuronal cell death. It has been previously shown^18^ that similarly to the drug treatment, cPLA2 KO mice are protected in SCI model. Our data indicate that AACOCF_3_ significantly reduces cPLA2 phosphorylation in the lysosomal fractions. Its known that AACOCF_3_ can also weakly inhibit sPLA2 and iPLA2, but with 4 orders of magnitude less potent than that on cPLA2^40^. In addition, C57/BL6 mice used in this study have a null mutation in the gene encoding sPLA2^41^, thus eliminating the possibility of its involvement in the lysosomal damage. While iPLA2 is expressed in the mouse CNS, its function is downregulated following trauma^42,43^. Thus, the protective effect of AACOCF_3_ in SCI mice is likely the result of inhibition of cPLA2, although we cannot exclude the possibility of additional off target effects. It has been previously^18^ shown that early inhibition of cPLA2 decreases lesion volume and increases spared white matter at 6 weeks post-SCI, suggesting that cytoprotective effects are sustained over time. In addition, phosphorylated cPLA2 has been reported to be expressed in oligodendrocytes and subsets of microglia/macrophages and astrocytes^17,19^. Whether or not cPLA2 activation participates lysosomal functions in these cells needs further investigation. If depletion of cPLA2 in knock out mice dramatically improve lysosomal function after SCI is intriguing for future study. Although both neurons and mature oligodendrocytes undergo apoptosis following SCI^1,2^, the peak of oligodendrocytes cell death occurs later around 3–7 days after SCI^44^. Therefore, majority of TUNEL+ cells observed in this study at 24 h should be neurons. In addition, our quantified data were from the ventral horns, which are enriched in neuronal cells. However, we cannot exclude some contribution from different cell types.

Together, our data confirm that autophagy flux is impaired after traumatic SCI, and the main cause of this event is loss of lysosomal function. Increased activity of cPLA2 and its translocation to the lysosome fraction are the important factors in the process of lysosomal damage after SCI and the subsequent impairment of autophagy flux, which in turn induce neuronal cell death in the spinal cord. We also show through application of the drug AACOCF_3_ that lysosomal damage, inhibition of autophagy, and neuronal cell death can be attenuated by inhibition of cPLA2 activity. Thus, we propose that inhibiting cPLA2-mediated lysosomal damage early after SCI may restore autophagosome clearance and decrease neuronal cell loss.

## Materials and methods

### Mouse spinal cord injury contusion model

Adult male C57/BL6 mice at 8–10 weeks old and 20–25 g body weight were obtained from Taconic. After being anesthetized with isoflurane, the subject mice received spinal contusions at T10 level using the Infinite Horizon Spinal Cord Impactor (Precision Systems and Instrumentation) with a force of 60 kdyn, a moderate injury^45–47^. Manual bladder expression was carried out at least three times daily until reflex bladder emptying was established at 7–14 days after SCI. For mice used as control animals, only laminectomy was performed after anesthesia. The number of mice at various time points in each study is indicated in the figure legends. All procedures were performed under protocols approved by the University of Maryland School of Medicine Institutional Animal Care and Use Committee (IACUC).

### Drug treatments

Sham and SCI mice were randomly assigned to a treatment group according to a randomized block experimental design. The PLA2 inhibitor, AACOCF_3_ (Enzo Life Sciences, US, Cat. No. BML-ST335) was dissolved in 100% ethanol and then diluted in a vehicle solution of 1% DMSO in normal saline. AACOCF_3_ was injected three times (1 h before injury, immediately following SCI and 4 h post injury) intraperitoneally in treatment group (*n* = 6) at a dose of 15 mg/kg based on prior investigation^17,18^. Mice of the control group (*n* = 6) were injected with an equal volume of ethanol diluted in the vehicle solution. Animals were anesthetized and processed for Western blot at 24 h after injury.

### Subcellular fractionation and lysosome enrichment process

Around 5-mm fragments of spinal cord tissue centered on the injury site or corresponding site in sham animals were collected from sham mice, and at 2 or 24 h after and homogenized in ice-cold buffered solution containing 0.32 M sucrose, 10 mM Hepes with the addition of protease and phosphatase inhibitors^24^. Homogenates were centrifuged at 800 × *g* for 10 min at 4 °C to pellet down the nuclei. Supernatants were sequentially centrifuged at 20,000 × *g* for 20 min at 4 °C to pellet the heavy membrane/crude lysosomal fractions and at 100,000 × *g* for 1 h at 4 °C to pellet light membrane fractions. Both supernatant and suspended pellet fractions were recentrifuged to minimize cross contamination from the different subcellular fractions. All pellets were resuspended in homogenization buffer. Protein concentration was estimated using BCA reagent; samples were analyzed by Western Blot.

For isolation of purified lysosome samples, after the 5 mm segment of the spinal cord was extracted, the lysosome enrichment kit (Thermo Scientific, Cat.) was used according to the manufacturer’s instructions to obtain a purified form of lysosome for downstream testing with Western Blot analysis and MASPEC lipidomic.

### Lysosomal activity assays

Following subcellular fractionation, a portion of the crude lysosomal fractions was used to for the testing of enzymatic activity. The CTSD and NAG assays were performed using a fluorometric assay kits (BioVisison, Cat. No. K143; Sigma-Aldrich, Cat. No. CS0780) as per the manufacturers’ instructions. Fluorescence released from the synthetic substrate was measured using a fluorescent plate reader (Synergy Hybrid, Biotek) at Ex/Em = 328/460 nm for CTSD activity and absorbance at 405 nm for NAG activity.

### cPLA2 activity assay

cPLA2 activity assay was performed with the cPLA2 Assay Kit (Cayman Chemicals, US, Cat. No. 765021) according to the manufacturer’s instructions. In brief, at 24 h after surgery, mice were perfused with normal saline to remove blood clots and a 5 mm segment of spinal cord tissue was collected from either the epicenter of SCI mice or the corresponding region of sham mice and was processed with the lysosome enrichment kit. After obtaining lysosome enriched samples, we resuspended the pellets in 50 μl Lysis Buffer and mixed the samples with assay buffer in accordance to the manual, with triplicate wells of each sample. The absorbance was measured with a plate reader (Synergy Hybrid, Biotek) at 414 nm. Protein concentration of each sample was determined via the Pierce BCA method and the results are represented as a ratio of (absorbance rate)/(μg of protein).

### Immunohistochemistry (IHC)

For IHC, animals were intracardially perfused with normal saline, followed by 4% paraformaldehyde. A 1.0 cm segment of spinal cord centered at the injury site or in approximate regions for sham mice was cut into coronal sections of 20 μm thickness and thaw-mounted onto Superfrost Plus slides (Thermo-Fisher). Sections were blocked with 5% goat or donkey serum diluted in 0.3% Triton X-100 solution and incubated overnight with primary antibodies. After a 2 h incubation period of the secondary antibodies, cell nuclei were labeled with 4′,6-diamidino-2-phenylinodole (DAPI, Sigma-Aldrich), slides were cover-slipped with an anti-fade medium (Hydromount, National Diagonistics). Primary antibodies used include: SQSTM1 (1:200; Progen, GP62-C), NeuN (1:500; Millipore, MAB377), CTSD/Cathepsin D (1:100, SantaCruz Biotechnology, sc-6486), CTSL (1:250; R&D Systems, AF1515 and MAB9521), phospho-cPLA2 (1:400; Sigma-Aldrich, SAB4503812), Lamp2 (1:100; GL2A7, developed by Granger, B.L. and obtained from Developmental Studies Hybridoma Bank, developed under the auspices of the NICHD and maintained by The University of Iowa, Department of Biology, Iowa City, IA 52242). Secondary antibodies: alexa fluor 488 goat anti-rabbit (A11034), Alexa fluor 546 anti-rabbit (A11035), alexa fluor 546 goat anti-mouse (A11030), alexa fluor 568 goat anti-guinea pig (A11075), alexa fluor 633 goat anti-mouse (A21052) and alexa fluor 546 donkey anti-goat (A11056) (Invitrogen), Cf633 donkey anti-rat (SAB4600133) and Cf633 donkey anti-guinea pig (SAB4600129) (Sigma-Aldrich). TUNEL assay was performed on frozen brain sections using ApopTag In Situ Apoptosis Detection Kit (Millipore, S7165) as per the manufacturer’s protocol.

### Image acquisition and quantification

All images were acquired 0.5–1 mm rostral to the epicenter. Images from ventral horns of gray matter were acquired using a fluorescent Nikon Ni-E upright microscope, at ×20 (CFI Plan APO VC 20× NA 0.75 WD 1 mm) magnification^11,12^. All images for each data set were acquired using the same parameters (magnification, exposure time, gain, etc). The background of each image was subtracted using background region of interest (ROI). All images were quantified in unbiased automated manner using custom macros in Elements: nuclei were identified using Spot Detection algorithm; cells positive for any of the immunofluoresece markers were identified using detect regional maxima algorithm, followed by global thresholding. Number of positive cells were normalized to the total imaged ventral horn area (mm^2^). Intracellular puncta were detected using Spot Detection and normalized to the number of cells imaged. For each experiment data from images from same region in each mouse was summed and used for final statistical analysis. At least 500–1000 cells were quantified per mouse per experiment. All quantification was performed on original unedited images. For visualization purposes in figures only brightness and contrast were adjusted; all adjustments were applied to entire image area and equally to all panels in the same figure. In multicolor overlay images brightness of the DAPI channel was selectively decreased to allow better visualization of other channels.

### Sample preparation and western blot analysis

Mice spinal cord tissue at a length of 5 mm were extracted from the epicenter of injury groups with either AACOCF3 or vehicle treatment were sacrificed at 24 h after SCI. For sham animals, an equal length of 5 mm was extracted at the same time from the approximate area of T10. For sample processing, all tissue samples were immersed in RIPA Lysis Buffer (Sigma-Aldrich) supplemented with 1 × protease inhibitor cocktail (Sigma-Aldrich, US), Phosphatase Inhibitor Cocktail II and Phosphatase Inhibitor Cocktail III (Sigma-Aldrich, US). The tissue samples were homogenized on ice, followed by sonication and centrifuged at 20,000 × *g* for 20 min. Protein concentration was determined by the Pierce BCA method (Thermo-Fisher Scientific, US). Samples were run on 4–20% SDS-PAGE (Bio-Rad, US) and transferred to 0.2-μm nitrocellulose membrane (Bio-Rad, US). Membranes were blocked with 10% nonfat skim milk in PBST, incubated overnight with primary antibodies diluted in blocking buffer and incubated for 2 h in HRP-conjugated secondary antibodies. After the immunoblots were visualized with SuperSignal West Dura Extended Duration Substrate (Thermo-Fisher Scientific, US) and imaged with ChemiDoc TM MP system (Bio-Rad, US), the optical density of signal bands was quantified by Image Lab software (Bio-Rad). Primary antibodies and respective dilution rates are as followed: LC3 (1:1000; Novus Biologicals, NB100-2220), p62/SQSTM1 (1:1000; BD Bioscience, 610832), β-actin (1:10,000; A1978), phospho-cPLA2 (1:1000; SAB4503812) (Sigma-Aldrich), LAMP1 (1:1000; Abcam, 24170), cPLA2 (1:1000; 2832), GAPDH (1:2000; Millipore, Cat. No. AB2302), and Fordin/SPTAN1 (1:5000; Enzo Life Science International, Cat. No. BML-FG6090),

### Lipidomics

Lipidomic analysis of lysosomal extracts has been described previously^25^. Briefly, total lipid extracts from the lysosome samples were prepared using MTBE lipid extraction protocol^48^ and analyzed using ultrahigh performance liquid chromatography coupled to data-independent tandem mass spectrometry coupled to traveling wave ion mobility (HDMS^E^). Liquid chromatography was performed on a Waters ACQUITY UPLC system (Milford, MA). The separation was achieved using a C18 CSH (1.7 µm; 2.1 × 100 mm) column. UPLC parameters were adopted with slight modifications from Damen et al.^49^ Mobile phase A was 10 mM ammonium formate with 0.1% formic acid in water/acetonitrile (40:60, v/v) and mobile phase B was 10 mM ammonium formate with 0.1% formic acid in acetonitrile/isopropanol (10:90, v/v). HDMS^E^ experiments were performed with a traveling wave ion-mobility enabled hybrid quadrupole orthogonal acceleration time-of-flight mass spectrometer (SYNAPT G2-S, Waters Corporation, Wilmslow, United Kingdom). HDMS^E^ parameters were adopted with slight modifications from Paglia et al.^50^ The instrument was operated in positive and negative ion mode electrospray. Data were acquired over the *m/z* range of 100–1800. The mass spectrometer was operated in ion mobility, data-independent acquisition for both positive and negative ion modes. The first scan was set at low-collision energy (4 eV) and used to collect precursor ion spectra. The second scan was set at high-collision energy and ramped from 30–55 eV, which was used for generation of product ion spectra. Argon gas was used for collision-induced dissociation (CID). Leucine Enkephalin (0.1 mg/mL) at a flow rate of 7.5 µL/min was used as the lock-mass to ensure high mass accuracy data acquisition. Poly-DL-alanine was used for collisional cross section calibration at a concentration of 10 µg/mL. Data were acquired with MassLynx v4.1 (Waters Corporation, Wilmslow, United Kingdom).

#### Data processing/bioinformatics

UHPLC-HDMS^E^ data were analyzed with MS^E^ Data Viewer v1.2 (Waters), DriftScope HDMS v2.7 (Waters), Progenesis QI v2.2 (Nonlinear Dynamics, Newcastle, United Kingdom), MetaboAnalyst 3.0 (Xia et al.), and Prism 6 (GraphPad, La Jolla, CA). Raw data files were directly imported into Progenesis QI where retention time alignment, peak picking, deconvolution of adducts, relative abundance, and preliminary identification were performed. Preliminary identification involved accurate mass correlation at a threshold of 10 ppm to LIPIDMAPS (http://www.lipidmaps.org). The processed data generated from Progenesis QI, which included peak area and *m*/*z* value, was exported into MetaboAnalyst for multivariate analysis. Multivariate analysis included principal component analysis (PCA) and partial least square discriminate analysis (PLS-DA). Univariate analysis via Prism 6 was performed using normalized values generated from Progenesis QI. Putative and confirmatory structure assignments relied on chromatographic retention time, HDMS^E^, positive and negative ion spectral correlation, and for a number of selected lipids authentic standard verification.

### Statistical analysis

All results are expressed as mean + SEM, where “*n*” represents the number of individual animals per group. The number of animals in all studies was determined by power analysis (power of 0.8 with alpha value 0.05). Key experiments were repeated with independent groups of animals to ensure reproducibility. All statistical analyses were conducted using SigmaPlot, Version 12 (Systat Software, San Jose, CA) or GraphPad Prism, Version 7.04 (GraphPad Software, La Jolla, CA). One-way ANOVA followed by Bonferroni, Tukey’s or SNK *t*-test post-hoc was used for parametric data. Kruskal–Wallis ANOVA based on ranks and Dunn’s post-hoc test was used for nonparametric data. For experiments with only two groups two-tailed unpaired Student’s *t*-test (parametric) was performed. A *p* value < 0.05 was considered to be significant.

## Supplementary information

Suppl Information

Suppl Figure 1

Suppl Table 1
