# Supplementary material for: cPLA2 activation contributes to lysosomal defects leading to impairment of autophagy after spinal cord injury
Source: Cell Death Dis. 2019 Jul 11;10(7):531. doi: 10.1038/s41419-019-1764-1 (PMC6624263; doi:10.1038/s41419-019-1764-1)
Supplement: Supplementary file 1 — Suppl Information [file 41419_2019_1764_MOESM1_ESM.docx]

**Supplementary Information:**

- **Supplementary Figure S1 (PDF)**
- **Supplementary Table S1 (PDF)**
