# Supplementary material for: cPLA2 activation contributes to lysosomal defects leading to impairment of autophagy after spinal cord injury
Source: Cell Death Dis. 2019 Jul 11;10(7):531. doi: 10.1038/s41419-019-1764-1 (PMC6624263; doi:10.1038/s41419-019-1764-1)
Supplement: Supplementary file 2 — Suppl Figure 1 [file 41419_2019_1764_MOESM2_ESM.pdf]

a

Lipidomic workflow

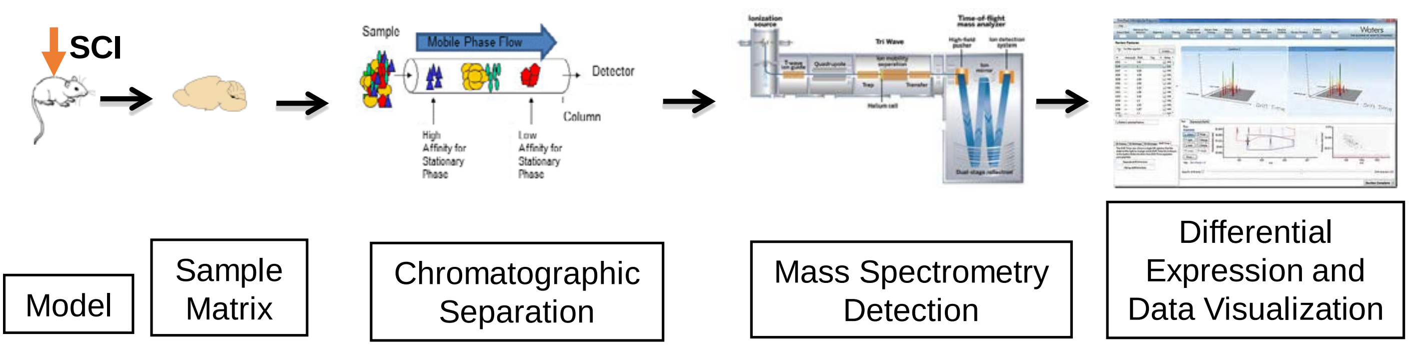

b

Phospholipids: PC, PE, PS, PG, SM

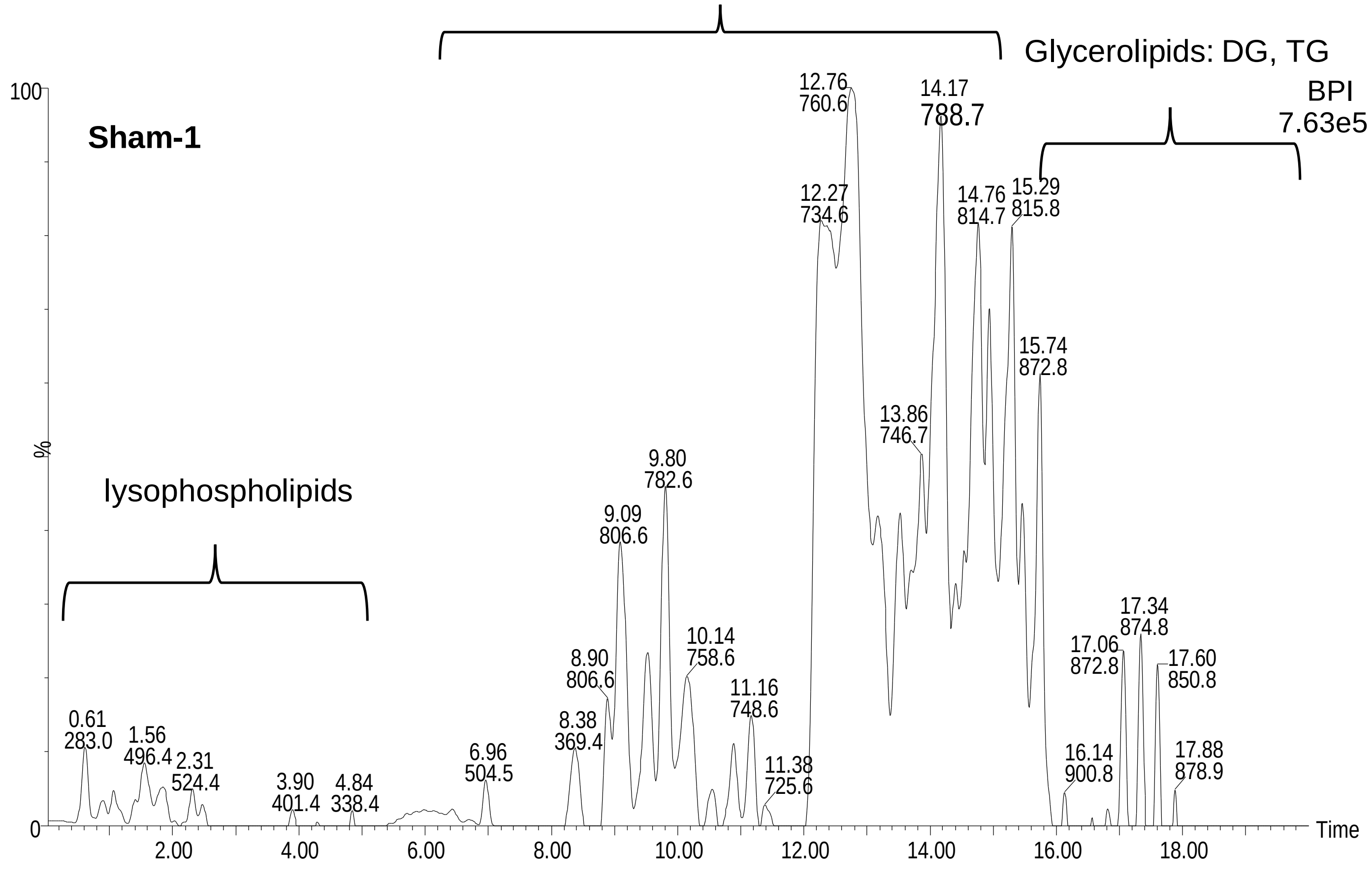

Supplementary Figure S1. Lipidomic analysis of lysosomal membranes by LC-MS. Related to Figure 3. (**a**) Lipidomics workflow representing how samples were collected, processed and analyzed to obtain lipid profiles of lysosomal membranes. (**b**) Example UPLC-HDMSE data for identification of LPC (18:0). Base peak ion chromatogram for entire chromatographic run (20 minutes), extracted ion chromatogram for m/z 524.3710, higher-collision energy mass spectrum for m/z 524.3710, and proposed structure for m/z 524.3710 are shown. Identification is based on chromatographic retention, accurate mass precursor ion, and ion mobility aligned tandem mass spectrometry.
