# Supplementary material for: cPLA2 activation contributes to lysosomal defects leading to impairment of autophagy after spinal cord injury
Source: Cell Death Dis. 2019 Jul 11;10(7):531. doi: 10.1038/s41419-019-1764-1 (PMC6624263; doi:10.1038/s41419-019-1764-1)
Supplement: Supplementary file 3 — Suppl Table 1 [file 41419_2019_1764_MOESM3_ESM.pdf]

Table S1. List comparing identified lipids differentially expressed between Sham and SCI (87 lipids)

	Input Mass	Matched Mass	Delta	Name	Anova (p)	q Value	FC	Highest Mean	Max Abun
1	281.2492	281.2486	0.0006	FA(18:1)	0.0102	0.0610	1.2	SCI	5997.5
2	309.2810	309.2799	0.0011	FA(20:1)	0.0090	0.0587	1.5	SCI	109.2
3	337.3117	337.3112	0.0005	FA(22:1)	0.0092	0.0587	1.5	SCI	39.8
4	365.3432	365.3425	0.0007	FA(24:1)	0.0490	0.1205	1.8	SCI	28.4
5	520.5090	520.5088	0.0002	Cer(d34:1)	0.0395	0.1661	1.3	SCI	2658.2
6	644.5993	644.5952	0.0041	Cer(d40:1)	0.0240	0.1461	2.8	SCI	947.1
7	672.6250	672.6265	0.0015	Cer(d42:1)	0.0247	0.1468	3.4	SCI	876.9
8	806.6507	806.6480	0.0027	HexCer(d40:1)	0.0277	0.1486	2.3	SCI	140898.6
9	834.6792	834.6793	0.0001	HexCer(d42:1)	0.0316	0.1526	2.9	SCI	348944.8
10	822.7249	822.7181	0.0068	HexCer(d44:1)	0.0278	0.1489	2.5	SCI	102.2
11	860.5953	860.5927	0.0026	SHexCer(d40:2)	0.0074	0.0552	1.2	SCI	2844.4
12	888.6258	888.6240	0.0018	SHexCer(d42:2)	0.0258	0.0911	1.1	SCI	102981.7
13	733.6205	733.6218	0.0013	SM(d36:0)	0.0112	0.1197	1.1	SCI	48039.1
14	761.6509	761.6531	0.0022	SM(d38:0)	0.0218	0.1424	1.4	SCI	56683.1
15	817.7077	817.7157	0.0080	SM(d42:0)	0.0424	0.1686	2.5	SCI	4320.8
16	815.6982	815.7000	0.0018	SM(d42:1)	0.0461	0.1722	2.0	SCI	148720.5
17	857.6778	857.6753	0.0025	SM(d42:2)	0.0314	0.0990	1.2	SCI	13973.4
18	496.3397	496.3398	0.0001	LPC(16:0)	0.0015	0.0798	1.4	SCI	34422.2
19	494.3229	494.3241	0.0012	LPC(16:1)	0.0457	0.1722	1.6	SCI	320.5
20	524.3674	524.3711	0.0037	LPC(18:0)	0.0029	0.0981	1.5	SCI	25188.2
21	520.3368	520.3398	0.0030	LPC(18:2)	0.0019	0.0843	2.0	SCI	865.1
22	550.3864	550.3867	0.0003	LPC(20:1)	0.0246	0.1468	1.3	SCI	4135.2
23	572.3679	572.3711	0.0032	LPC(22:4)	0.0207	0.1408	1.6	SCI	343.0
24	482.3600	482.3605	0.0005	LPC(O-16:0)	0.0059	0.1051	1.5	SCI	314.7
25	480.3436	480.3448	0.0012	LPC(P-16:0)	0.0264	0.1474	1.6	SCI	473.4
26	508.3756	508.3761	0.0005	LPC(P-18:0)	0.0330	0.1545	1.8	SCI	240.2
27	758.5694	758.5694	0.0000	PC(34:2)	0.0310	0.1519	1.1	SCI	29952.8
28	756.5494	756.5538	0.0044	PC(34:3)	0.0238	0.1461	1.3	Sham	79917.3
29	754.5350	754.5381	0.0031	PC(34:4)	0.0058	0.1045	1.5	Sham	4444.9
30	782.5648	782.5694	0.0046	PC(36:4)	0.0335	0.1554	2.0	Sham	956.2
31	810.5991	810.6007	0.0016	PC(38:4)	0.0273	0.1482	1.3	Sham	50851.8
32	804.5503	804.5538	0.0035	PC(38:7)	0.0089	0.1126	1.9	SCI	565.0
33	838.6275	838.6320	0.0045	PC(40:4)	0.0325	0.1535	2.0	SCI	4922.6
34	834.5969	834.6007	0.0038	PC(40:6)	0.0198	0.1408	1.7	Sham	377.8
35	830.5633	830.5694	0.0061	PC(40:8)	0.0043	0.0995	1.8	Sham	430.1
36	828.5515	828.5538	0.0023	PC(40:9)	0.0049	0.1006	2.1	Sham	195.9
37	866.6590	866.6633	0.0043	PC(42:4)	0.0014	0.0786	1.2	Sham	28364.0
38	864.6439	864.6477	0.0038	PC(42:5)	0.0143	0.1323	1.3	Sham	9058.4
39	862.6228	862.6320	0.0092	PC(42:6)	0.0154	0.1345	1.5	Sham	565.3
40	856.5775	856.5851	0.0076	PC(42:9)	0.0425	0.1689	2.0	Sham	149.6
41	892.6744	892.6790	0.0046	PC(44:5)	0.0061	0.1055	1.1	Sham	13502.5
42	746.5995	746.6058	0.0063	PC(O-34:1)/PC(P-34:0)	0.0450	0.1722	1.1	Sham	63919.3
43	772.6163	772.6215	0.0052	PC(O-36:2)/PC(P-36:1)	0.0099	0.1126	1.7	SCI	46.6
44	770.6050	770.6058	0.0008	PC(O-36:3)/PC(P-36:2)	0.0407	0.1668	1.2	SCI	9015.5
45	798.6301	798.6371	0.0070	PC(O-38:3)/PC(P-38:2)	0.0142	0.1321	4.2	SCI	38.6
46	796.6159	796.6215	0.0056	PC(O-38:4)/PC(P-38:3)	0.0402	0.1661	2.1	SCI	147.9
47	830.6920	830.6997	0.0077	PC(O-40:1)/PC(P-40:0)	0.0237	0.1460	2.0	SCI	4990.7
48	826.6640	826.6684	0.0044	PC(O-40:3)/PC(P-40:2)	0.0407	0.1668	1.5	SCI	3009.4
49	820.6144	820.6215	0.0071	PC(O-40:6)/PC(P-40:5)	0.0383	0.1653	1.5	SCI	5435.9
50	848.6518	848.6528	0.0010	PC(O-42:6)/PC(P-42:5)	0.0324	0.1532	2.2	SCI	20303.6
51	452.2794	452.2783	0.0011	LPE(16:0)	0.0019	0.0328	1.6	SCI	3539.8
52	450.2625	450.2626	0.0001	LPE(16:1)	0.0081	0.0558	5.1	SCI	57.0
53	478.2956	478.2939	0.0017	LPE(18:1)	0.0239	0.0886	1.4	SCI	28995.9
54	476.2781	476.2783	0.0002	LPE(18:2)	0.0370	0.1049	3.5	SCI	27.9
55	500.2796	500.2783	0.0013	LPE(20:4)	0.0301	0.0979	1.9	SCI	1607.7
56	528.3104	528.3096	0.0008	LPE(22:4)	0.0196	0.0821	1.7	SCI	1984.9
57	468.3443	468.3448	0.0005	LPE(O-18:0)	0.0312	0.1520	1.3	SCI	98.7
58	466.3280	466.3292	0.0012	LPE(P-18:0)	0.0355	0.1595	2.4	SCI	48.6
59	708.4503	708.4599	0.0096	PE(34:6)	0.0359	0.1602	4.5	SCI	3704.4
60	742.5417	742.5392	0.0025	PE(36:2)	0.0461	0.1169	1.1	SCI	12232.6
61	738.5070	738.5079	0.0009	PE(36:4)	0.0465	0.1169	15.8	SCI	26.0
62	738.5066	738.5068	0.0002	PE(36:5)	0.0188	0.1399	1.6	SCI	71.4
63	792.5521	792.5538	0.0017	PE(40:6)	0.0471	0.1738	1.3	Sham	168526.7
64	860.7170	860.7103	0.0067	PE(44:0)	0.0389	0.1660	2.3	SCI	8046.5
65	846.6078	846.6007	0.0071	PE(44:7)	0.0284	0.1502	2.2	Sham	81.4
66	730.5736	730.5745	0.0009	PE(O-36:2)/PE(P-36:1)	0.0078	0.1111	1.1	Sham	607694.0
67	782.5966	782.6058	0.0092	PE(O-40:4)/PE(P-40:3)	0.0229	0.1460	2.7	Sham	503.8
68	778.5762	778.5756	0.0006	PE(O-40:5)/PE(P-40:4)	0.0307	0.0983	1.1	Sham	11210.7
69	778.5737	778.5745	0.0008	PE(O-40:6)/PE(P-40:5)	0.0201	0.1408	1.2	Sham	104393.5
70	776.5544	776.5589	0.0045	PE(P-40:6)	0.0307	0.1519	1.6	Sham	904.9
71	773.5387	773.5338	0.0049	PG(36:2)	0.0022	0.0346	1.9	SCI	185.8
72	859.6482	859.6434	0.0048	PG(42:1)	0.0210	0.0861	47.6	SCI	24.5
73	816.5772	816.5760	0.0012	PS(38:1)	0.0280	0.0940	2.3	SCI	235.9
74	844.6100	844.6073	0.0027	PS(40:1)	0.0122	0.0669	1.1	SCI	4427.8
75	896.6331	896.6386	0.0055	PS(44:3)	0.0474	0.1179	2.0	Sham	66.5
76	641.5093	641.5115	0.0022	DG(36:3)	0.0440	0.1710	3.3	SCI	45.6
77	689.5164	689.5115	0.0049	DG(40:7)	0.0298	0.1510	2.0	Sham	687.2
78	877.6605	877.6680	0.0075	DG(54:11)	0.0207	0.1408	1.7	SCI	11.2
79	900.7440	900.7439	0.0001	DG(56:11)	0.0308	0.1519	1.5	SCI	75.1
80	789.5974	789.6004	0.0030	TG(46:6)	0.0213	0.1412	1.4	Sham	2940.3
81	787.5936	787.5847	0.0089	TG(46:7)	0.0239	0.1461	1.5	Sham	16806.5
82	868.7324	868.7389	0.0065	TG(52:6)	0.0268	0.1478	1.4	SCI	442149.0
83	977.7547	977.7569	0.0022	TG(60:10)	0.0320	0.1526	1.5	SCI	478.0
84	1052.8705	1052.8641	0.0064	TG(66:12)	0.0018	0.0836	2.0	SCI	5000.0
85	1089.8781	1089.8821	0.0040	TG(68:10)	0.0293	0.1505	5.9	SCI	44.6
86	1080.8993	1080.8954	0.0039	TG(68:12)	0.0010	0.0761	2.7	SCI	676.4
87	1078.8838	1078.8797	0.0041	TG(68:13)	0.0013	0.0782	2.5	SCI	264.2

Supplementary Table S1. There was a total of 87 lipids identified. Identification was based on UPLC HDMS^{E} as described previously[25]. Delta refers to mass accuracy in ppm (parts per million). FA = fatty acyl, Cer = ceramide, HexCer = hexosylceramide, SM = sphingomyelin, LPC = lysophosphatidylcholine, PC = phosphatidylcholine, LPE = lysophosphatidylethanolamine, PE = phosphatidylethanolamine, PG = phosphatidylglycerol, PS = phosphatidylserine, DG = diacylglycerol, and TG = triacylglycerol. FC = fold change.
